# 1236. Baseline liver stiffness and alanine transaminase predicts reduction in liver stiffness in people with chronic hepatitis B on tenofovir alafenamide

**DOI:** 10.1093/ofid/ofac492.1068

**Published:** 2022-12-15

**Authors:** Lydia Tang, Nivya George, Angie Price, Shyam Kottilil

**Affiliations:** Institute of Human Virology, University of Maryland school of medicine, Baltimore, Maryland; University of Maryland School of Medicine, Baltimore, Maryland; University of Maryland school of medicine, Baltimore, Maryland; Institute for Human Virology (IHV), University of Maryland School of Medicine, Baltimore, Maryland

## Abstract

**Background:**

Globally, 296 million people are infected with chronic hepatitis B (CHB) and at risk for cirrhosis and hepatocellular carcinoma. Suppressive therapy with a nucleos(t)ide analogue (NUC) such as tenofovir alafenamide (TAF) reduces risk of cirrhosis and cancer. Transient elastography measures liver stiffness and is used to monitor liver fibrosis in CHB. This study evaluates liver stiffness change among people taking NUC and determines correlates with improvement in liver stiffness.

**Methods:**

Participants enrolled at the University of Maryland, Baltimore were treated with TAF 25mg daily for 2 years. Liver stiffness (FibroScan©) was measured at baseline and year 2. Mild (F0/F1) and advanced (≥ F2) fibrosis, was defined as ≤ 7.4kPa and ≥ 7.5kPa, respectively. Correlates of liver stiffness change from baseline to year 2 was evaluated with multiple linear regression. P-value of < 0.05 was considered statistically significant.

**Results:**

Since 2017, 60 have completed the study. Twenty-nine were switched from another NUC to TAF and 31 were off NUC treatment at baseline (“No NUC”: 17 treatment naïve, 14 prior NUC). Baseline alanine transaminase (ALT) was higher in the "No NUC" group (53.5 IU/ml ± 42.7 versus 30.2 IU/ml ± 15.5, p = < 0.007). Most (67.2%) participants had mild fibrosis at baseline. Nineteen had advanced fibrosis. Among those with advanced fibrosis, fibrosis stage improved by at least 1 stage in 14 with no change in 5. Liver stiffness improved in the No NUC group (mean change in stiffness -1.2161 kPa, p = 0.02), but not in the switch group. Subgroup analysis of those with advanced fibrosis also showed reduction in liver stiffness in the No NUC group only. Increased baseline ALT and baseline liver stiffness were predictive of reduction in liver stiffness (rho= - 0.3, p= 0.005 and rho= - 0.7, p < 0.0001, respectively).

Baseline liver stiffness predicts change in liver stiffness with NUC therapy

**Figure:**
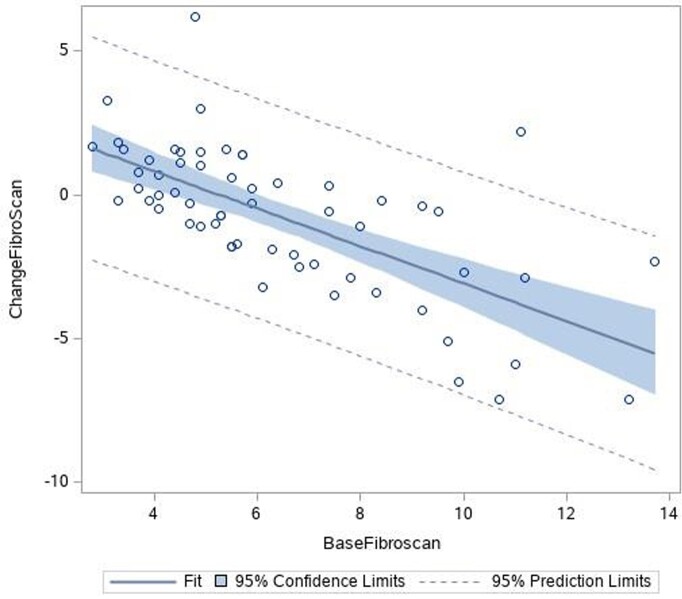

Increased liver stiffness, and therefore fibrosis, at baseline was associated with greater reductions in liver stiffness, and therefore liver stiffness, after 2 years of NUC therapy (rho = -0.7, p< 0.0001).

**Conclusion:**

Liver stiffness improved with 2 years of NUC treatment in people with CHB, including those with advanced liver fibrosis. Either baseline ALT or liver stiffness can predict reduction in liver stiffness. This may reflect an overestimation of fibrosis stage in the presence of liver inflammation at NUC initiation. Repeating FibroScan after initiation of NUC treatment would therefore be useful, especially in people with elevated ALT, for guiding CHB care.

**Disclosures:**

**Lydia Tang, MBChB**, Gilead Sciences: Grant/Research Support **Shyam Kottilil, MD, PhD**, Arbutus Pharmaceuticals: Grant/Research Support|Gilead: Grant/Research Support|Merck: Grant/Research Support|Regeneron Pharmaceuticals: Advisor/Consultant|Silverback Therapeutics: Advisor/Consultant|The Liver Company: Advisor/Consultant|Yufan Biotechnologies: Advisor/Consultant.

